# Open-access database of literature derived drug-related *Torsade de Pointes* cases

**DOI:** 10.1186/s40360-021-00548-0

**Published:** 2022-01-10

**Authors:** Laura Krumpholz, Barbara Wiśniowska, Sebastian Polak

**Affiliations:** 1 Pharmacoepidemiology and Farmacoeconomics Unit, Faculty of Pharmacy, Jagiellonian University Medical College, Medyczna 9, str., 30-688 Krakow, Poland; 2 Certara UK Ltd. (Simcyp Division), 1 Concourse Way, Sheffield, S1 2BJ UK

**Keywords:** Torsade de Pointes, TdP, Drug-induced TdP, Proarrhythmic potential, Drug cardiotoxicity, Database

## Abstract

Since an introduction of an ICH guidance in 2005, no new drugs were withdrawn from the market because of the causation of Torsade de Pointes (TdP). However, the risk of TdP is still a concern for marketed drugs. TdP is a type of polymorphic ventricular tachycardia which may lead to sudden cardiac death. QT/QTc interval prolongation is considered a sensitive, but not specific biomarker. To improve the effectiveness of studies’ workflow related to TdP risk prediction we created an extensive, structured, open-access database of drug-related TdP cases. PubMed, Google Scholar bibliographic databases, and the Internet, via the Google search engine, were searched to identify eligible reports. A total of 424 papers with a description of 634 case reports and observational studies were included. Each paper was manually examined and listed with up to 53 variables related to patient/population characteristics, general health parameters, used drugs, laboratory measurements, ECG results, clinical management, and its outcomes, as well as suspected drug’s properties and its FDA adverse reaction reports. The presented database may be considered as an extension of the recently developed and published database of drug cardiac safety-related information, part of the tox-portal project providing resources for cardiac toxicity assessment.

**Supplementary Information:**

The online version contains supplementary material available at 10.1186/s40360-021-00548-0.

## Background

The total probability of achieving the FDA approval for a new drug candidate in phase 1 of clinical trials is estimated as under 10% [1]. In 2000 drug attrition rate from toxicity and clinical safety reasons was estimated at approximately 30%. One of the main reasons for the withdrawals was the occurrence of safety liabilities relating to the cardiovascular system. Moreover, the most frequently reported post-approval cardiac adverse events were cardiac arrhythmias [2]. Given that cardiac issues, including QT prolongation/Torsade de Pointes (TdP) occurrence, were one of the major safety challenges between 1990-2006, studies related to the proarrhythmic risk of drugs became of high concern [3]. In 2005 two guidance documents on nonclinical (S7B) and clinical (E14) evaluation of cardiac risk were issued by the International Council for Harmonisation of Technical Requirements for Pharmaceuticals for Human (ICH) [4, 5]. Since then and after 2006 no new drugs were withdrawn from the market because of the causation of the TdP [6]. Despite being criticized for the conservative character, posing risk for unnecessary termination of potentially useful drug candidates because of the positive results for the hERG channel blocking liability test, those guidances fulfilled their intended purpose [7]. Nevertheless, the cases of TdP continue to occur for the marketed drugs with acknowledged proarrhythmic liability and are being reported in the literature. Also, several newly marketed drugs have been added to the QTdrugs list in the Possible Risk of TdP category by CredibleMeds research and education center [8].

Typically, the TdP event requires the simultaneous occurrence of several risk factors modifying individual risk including baseline QT prolongation, female sex, advancing age, genetic predisposition, electrolyte disturbance, heart diseases, or drug-drug interactions [3, 9, 10]. Therefore, this work aimed to develop a database of drug-induced TdP occurrence cases reported in the literature together with a detailed description of possible predisposing factors. Although there are available lists of compounds that are believed to prolong QT and increase the risk of developing TdP, such as CredibleMeds QT Drugs List, CiPA list with 27 drugs assigned to categories of high/intermediate/low risk of TdP, or Drug-Induced QT Prolongation Atlas (DIQTA) that presents overall characteristics of patients with prolonged reported to FDA Adverse Events Reporting System (FAERS) [11], we did not find any databases containing the detailed individual case description i.e. listing demographic and clinical details [12]. To develop a database that can be utilized e.g., for verification of TdP risk assessment models we decided to include results of QT measurement, basic biochemical measurements as well as a dose of the suspected drugs, whenever feasible.

The provided dataset may be utilized for more effective verification of in silico models developed to predict drugs TdP risk or analysis of the effects/influence of e.g., disease state, electrolyte status, drug-drug interactions on TdP risk alteration. Since TdP management strategy and patient outcomes were included, the database may be useful for analysis of the effectiveness of the QTc prolongation and TdP treatment. The presented database may be considered as an extension of the recently published database on TQT trials results [13].

## Construction and content

The premise for the presented database was to provide an extensive repository for the TdP incidences related to the use of drugs. It consists of the description of case studies and observational studies available in the literature. All data were structured in one Microsoft Excel table containing 53 parameters described below.

The database is stored as an Excel file and available in the Additional files section. It is also freely available on the tox-portal website [14] as part of a collection of databases on cardiac safety (including TQT studies, ion channels inhibition potential, drug-drug interactions resulting in QT prolongation) [15, 16].

Data gathering was performed between December 2020 and June 2021. PubMed, Google Scholar bibliographic databases, and the Internet, via the Google search engine, were searched to identify papers and reports describing case reports of the TdP occurrence. Searching was focused on a wide description of patients' state during admission to the hospital, emergency department, or psychiatric ward, through applied therapies, up to a brief description of planned or implemented follow-up procedures. To identify as many papers as possible different combinations of keywords were used. The search queries included the following keywords: “Torsade de Pointes”, “TdP”, “torsades”, “drug-related”, “drug-induced”, “cardiotoxicity”, “arrhythmia”. Case reports and observational studies reporting TdP occurrence were considered eligible. For each included paper ‘Similar articles’ PubMed functionality that provides additional, closely related citations was used. In the case of reviews, original sources were found, if feasible, and cases were described accordingly.

All the case details were extracted manually from relevant publications into a standardized Excel spreadsheet. First, a full text of a paper was carefully read, next, the second read was performed by extracting applicable data to the Excel file. To ensure quality, the same procedure was subsequently performed by another team member. The extracted data were compered, double-checked, missing or invalid data were completed and corrected or completed. In the case of studies where the units of lab measurements were not given, and it was not possible to unambiguously settle the correct one, the note that the units were not given was added. Missing data were left blank in the dataset.

The presented database includes 424 published papers, most of them published in English-language journals, with a detailed description of a total of 634 reports. The summary can be found in Table 1. Only one record reported clinical trials, and two were case series while the rest were individual case reports. The studies were published between 1980 and 2021. Twenty-five of the described cases were fatal. In 609 cases, patients were adults (over 18-years-old), including 237 elderly patients (over 65-years-old). In total over 63% of patients were female. Collated publications were related to TdP induced by 140 different drugs and toxins. The QT and/or QTc interval duration was specified in 565 cases and the QRS interval duration in 81 cases. The majority of cases (over 81%) concerned diseased individuals, and in 397 cases other drugs were taken concomitantly. Twenty-six cases resulted from a suicide attempt with single or multiple drugs overdosed. Overdosing cases (in total 67) were also a result of drug abuse (mostly loperamide – 24 cases) and accidental ingestion of a drug or toxin. About 70% of cases included a description of applied clinical management of TdP arrhythmia, however, post-therapy QRS and/or QT/QTc measurements were rarely provided (in 13 and 233 cases, respectively). Most of the cases were associated with electrolyte disturbance and required electrolyte replacement. In 393 cases plasma potassium concentration was given with 143 cases of hypokalemia therein (potassium concentration lower than 3.5 mmol/L). In some cases, discontinuation of the drug(s) suspected to induce QTc interval prolongation was a sufficient intervention to achieve the patient’s recovery.

**Table 1:** General characteristic of the cases included in the dataset

Characteristic	Number of relevant cases (% of total)	Number of cases where data were not available (% of total)
Total number of cases	634 (100%)	-
Adults (> 18-yr-old)	609 (96%)	6 (1%)
Elderly (> 65-yr-old)	237 (37)	6 (1%)
Female	398 (63%)	17 (3%)
Hypokaliemia was confirmed	143 (23%)	241 (38%)
More than one drug was reported	397 (63%)	-
Overdose	67 (11%)	-
Suicide attempt	23 (4%)	-
Drug abuse	24 (4%)	-
Concomitant alcohol ingestion	24 (4%)	-

Every case record contains information that enables the reference paper or report identification. The collated cases description was intended to provide clinical parameters relevant for the TdP risk assessment [17]. All presented papers and reports were examined individually for source information about patient or population characteristics (age, sex), patient’s general health parameters (comorbidity, history of drug-induced TdP), used drugs (polytherapy, overdosing), laboratory measurements (e.g. electrolyte imbalance), ECG results (e.g. QT/QTc interval length). Additionally, case clinical management, and its outcomes were included. Any other case-specific information is given as a comment. The database also includes basic information about the main suspected drug such as its phys-chem properties obtained from SwissADME tool developed by Swiss Institute of Bioinformatics [18], and a number of reports related to the drug in the FAERS (data as of March 31, 2021) [19]. Each study was identified with the first author surname, date of publishing, and PubMed unique identifier number (PMID). The cases were described by up to 53 parameters, which are presented in Table 2.

**Table 2:** Parameters used to describe cases in the dataset

Parameter Group	Parameter Name
Case ID	unique case identified
Source	author, year, PMID
Patient/population characteristic	age, sex, diseases
Patient’s general health parameters	blood pressure, heart rate, breaths (respiratory rate), temperature
Drugs and alcohol intake	main suspected drug (name, dose, route of administration, additional dosing information, therapeutic range), concomitant drugs (name, dose, route of administration, additional dosing information), overdose (y/n), alcohol
Lab measurements	blood pH, creatinine kinase, blood urea nitrogen, the plasma concentration of: main suspected drug, sodium, potassium, magnesium, calcium, chloride, bicarbonate, lactic acid
ECG parameters	QRS, QT, QTc interval duration
Clinical management and outcome	Applied clinical TdP management, post-intervention QRS, QT/QTc, outcome
Comments	Additional comments
Drug’s properties	SMILES, molecular weight, total polar surface area, logP, H-bond acceptors, H-bond donors, number of rotatable bonds, number of heavy atoms
FDA reports related to the suspected drug	Total number of reports, number of cardiac cases, number of TdP cases, deaths due to TdP

### Utility and discussion

The current project aimed to collect available data describing drug-triggered TdP cases, prepare a database and make it freely available. With that, we want to contribute to the drugs’ proarrhythmic potency screening efforts. This is needed for newly developed and already existing drugs and drug combinations. The latter, namely drug combinations are necessary to effectively treat various diseases, however, polytherapy can increase cardiotoxicity risk as there is a case for malaria [20]. Building an in silico model TdP risk assessment model was not an aim of the current study, however, we envisage the potential use of the above-described database for such endeavors e.g. the collection can be used for verification and assessment of the predictive performance of a model accounting for several TdP risk factors. It is worth mentioning that the database will be further curated, and new records will be added regularly.

## Conclusions

To the best of our knowledge, the presented set of data is the only publicly available collection of the literature-reported TdP case studies. The database is curated and freely available for all interested parties (Additional file 1 or tox-portal website [14]) This, in our opinion, includes clinicians and drug development/drug safety specialists. The drug-related arrhythmias are considered drug-specific although it is rather a combination of the drug itself (e.g. affinity to ion channels, DDIs) and physiological (e.g. sex, ions concentration in plasma) parameters. For the latter of the above-listed groups, namely drug development specialists, a systematic and consistent set of data can be potentially useful to distinguish between these two different groups of parameters.

## Supplementary Information

**Additional file 1: Excel file S1.** An Excel file of the database of the literature-derived drug-related Torsade de Pointes cases.

## Data Availability

All data generated or analysed during this study are included in this published article [and its supplementary information files].

## Abbreviations

ECG: Electrocardiography
FAERS: FDA adverse events reporting system
FDA: Food and drug administration
hERG: Potassium voltage-gated channel subfamily H member 2
ICH: International council for harmonisation
TdP: Torsade de Pointes
TQT: Thorough QT/QTc
