# Correction to: Open-access database of literature derived drug-related Torsade de Pointes cases

**DOI:** 10.1186/s40360-022-00550-0

**Published:** 2022-01-31

**Authors:** Laura Krumpholz, Barbara Wiśniowska, Sebastian Polak

**Affiliations:** 1 Pharmacoepidemiology and Farmacoeconomics Unit, Faculty of Pharmacy, Jagiellonian University Medical College, Medyczna 9, str., 30-688, Krakow, Poland; 2 Certara UK Ltd. (Simcyp Division), 1 Concourse Way, Sheffield, S1 2BJ UK

**Correction to: BMC Pharmacol Toxicol 23, 7 (2022)**

**https://doi.org/10.1186/s40360-021-00548-0**

Following publication of the original article [1], the authors identified an error in the authors names. The given names and family names were erroneously transposed.

The incorrect authors’ name are:

Given name: Krumpholz

Family name: Laura

Given name: Wiśniowska

Family name: Barbara

Given name: Polak

Family name: Sebastian

The correct authors names are:

Given name: Laura

Family name: Krumpholz

Given name: Barbara

Family name: Wiśniowska

Given name: Sebastian

Family name: Polak

The author group has been updated above and the original article [1] has been corrected.
